# Inert 37-Year-Old Intrastromal Corneal Graphite From Penetrating Ocular Trauma: A Case Report

**DOI:** 10.7759/cureus.50030

**Published:** 2023-12-06

**Authors:** Orwa Nasser, Minal Patil

**Affiliations:** 1 Ophthalmology, Orasis Eyecare Center, Ma'alot-Tarshiha, ISR; 2 Ophthalmology, Hadassah Academic College, Jerusalem, ISR; 3 Ophthalmology, Drishti Eye Institute, Dehradun, IND

**Keywords:** graphite, penetrating ocular trauma, ocular graphite., pencil injury, corneal trauma

## Abstract

Ocular trauma is the most frequent cause of monocular blindness. Ocular surface injury is the commonest among ocular injuries. Corneal abrasion and foreign body are the most common corneal injuries.

A 40-year-old dentist with a history of partially penetrating corneal injury due to a pencil presented with and retained graphite bodies in the cornea 37 years after injury. His refraction was +1.0 sphere with no astigmatic error. There was no evidence of any inflammatory reaction. Intrastromal corneal graphite particles can be inert and cause no inflammatory reaction or astigmatic error even decades after injury.

## Introduction

Ocular injuries are common reasons for patients to seek consultation in the emergency department [1]. Ocular trauma is the most frequent cause of noncongenital monocular blindness in children and adults [1]. The severity of vision loss in ocular trauma depends on the type, location, duration, and amount of injury [2].

The ocular surface injury is the commonest among ocular injuries as it is the exposed region of the eye. Corneal injuries can occur secondary to traumatic, chemical, inflammatory, metabolic, autoimmune, and iatrogenic causes [3]. Corneal abrasion and foreign body are the most common superficial corneal injuries [4].

In this case report, we present a rare condition of a partially penetrating corneal injury due to pencil and retained graphite bodies in the cornea 37 years after injury. This is the first case of retained graphite in cornea to be reported by clinical imaging and to be reported after so many years without ocular reaction, making it the case of intrastromal graphite with the longest duration without any evidence of reaction.

## Case presentation

**Figure 1:**
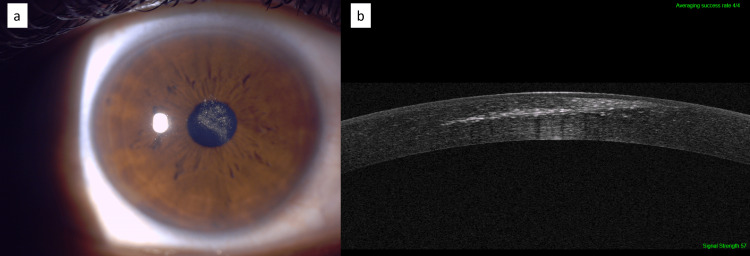
Graphite particles in the cornea 37 years after penetrating trauma Intrastromal refractile graphite particles (a). Anterior segment optical coherence tomography (ASOCT) shows a stromal refractile linear scar and a healed entry wound from the old injury with pencil (b).

A 40-year-old dentist presented with a complaint of reduced vision in the right eye since childhood. The patient reported an injury to the right eye with a pencil at the age of three years, which was treated with topical antibiotics and lubricants. His Best corrected visual acuity (BCVA) in the right eye was 20/25 and in the left eye was 20/20. His manifest refraction +1.0 sphere in both eyes. A slit lamp examination of the right eye showed a central linear corneal scar with refractile pigmentation involving the visual axis which extended into the anterior stroma of the cornea (Figure 1a). The corneal exam showed no evidence of any inflammatory reaction or any corneal scars or signs of previous corneal or stromal inflammation. The rest of the anterior segment exam and posterior segment exam showed no pathology. Anterior segment optical coherence tomography (ASOCT) showed a stromal pigmentation extending from the subepithelial layer to the mid-stromal layer of the cornea along with a linear scar in the mid-stroma (Figure 1b). ASOCT also revealed the presence of a healed entry wound from the old injury with a pencil (Figure 1b). These findings suggest that the corneal deposits are the deposition of graphite particles in the stroma which are residue from pencil tip penetrating trauma. Corneal tomography showed no significant corneal astigmatism or irregularities. The patient was treated by only optical correction with glasses according to manifest refraction. His BCVA was 20/25. No other intervention was performed and debridement of graphite particles from the cornea was not considered. Three months later, no change with the ocular exam was seen and the patient was happy with his optical correction by glasses.

## Discussion

Retained intraocular graphite foreign bodies are uncommon. However, various ocular sites were reported where graphite was noted including eyelid, intra-orbital, cornea, conjunctiva, anterior chamber, lens, and posterior segment [5-10].

The basic components of pencil lead are 66% graphite, 26% aluminum silicate, and 8% paraffin [11]. The white paraffin can cause nonallergic reactions however the most common reaction to lead is a delayed granulomatous reaction from graphite or aluminum silicate [12]. This delayed reaction is due to the slow breakdown of graphite pencil lead into small pieces which disperse and cause the release of T cells and macrophages which release cytokines and growth factors. Silica and carbon leads to granulomatous reaction while organic wood can lead to abscess formation and fungal infection [13,14].

Intrastromal graphite is inert, however, graphite in the anterior chamber or vitreous can cause delayed reaction [6-8]. The delayed reaction to graphite could be due to the occurrence of some triggering factor like trivial trauma which results in inflammatory cell migration and reaction. The inertness of intrastromal graphite can be attributed to the fact that in case of any corneal injury, the inflammation is confined to a region of injury due to compartmentalization of the reaction.

The presence of graphite in the anterior chamber has been shown to produce inflammatory reactions. However, it was shown that the reaction occurred after 20 years of injury in a case reported by Amy et al. [8]. This reaction to anterior chamber graphite presented as anterior chamber cells and keratin precipitates along with corneal edema at the site of the graphite foreign body. Similarly, in a case presented by Han et al., the anterior chamber graphite reaction masqueraded as viral stromal keratitis after 12 years of injury with a lead pencil [9]. The pencil lead in the vitreous cavity has been shown to cause sterile inflammatory reactions presumably due to the aluminum component in pencil lead [6].

Optic neuropathy along with strabismus was reported in the case of trauma with a pencil in which the lead was a peasant in the intra-orbital region. This led foreign body having an inflammatory reaction that caused compression of the optic nerve and medial rectus muscle [15]. The management of lead graphite foreign body with reaction is the removal of graphite lead along with systemic or topical steroids depending on the severity of the reaction [16].

However, prior studies on intrastromal graphite particles and penetrating damage have recommended against treating the intrastromal graphite particles while surgically treating the penetrating lesion. Leaving the intrastromal graphite particle has not shown to have any long-term complications [17].

In our case the intrastromal graphite lead was present, and a track of an entry wound was noticed on ASOCT. The injury was 37 years before presentation, making it the case of intrastromal graphite with the longest duration and without any evidence of reaction. Moreover, the presence of these particles did not cause any astigmatic error.

Previous case reports also showed the intrastromal graphite particles which were inert, and the history of injury was old. Most of the cases of intrastromal graphite were incidentally diagnosed. As these cases had no evidence of reaction, no intervention was done for the graphite particles.

## Conclusions

In conclusion, the intrastromal graphite particles can be diagnosed incidentally, usually inert, and can cause no inflammatory reaction decades after injury. Graphite particles in the cornea might not cause any astigmatic error.
